# Worldwide Trends in Survival From Common Childhood Brain Tumors: A Systematic Review

**DOI:** 10.1200/JGO.19.00140

**Published:** 2019-11-04

**Authors:** Fabio Girardi, Claudia Allemani, Michel P. Coleman

**Affiliations:** ^1^ London School of Hygiene and Tropical Medicine, London, United Kingdom

## Abstract

**PURPOSE:**

The histology of brain tumors determines treatment and predicts outcome. Population-based survival reflects the effectiveness of a health care system in managing cancer. No systematic review of worldwide variation and time trends in survival from brain tumors in children is currently available.

**PATIENTS AND METHODS:**

We considered longitudinal, observational studies comprising children diagnosed with intracranial astrocytic or embryonal tumors. We searched six electronic databases from database inception to September 30, 2018, using complex search strategies. The outcome measure was 5-year survival, estimated through a time-to-event analysis. This study is registered with PROSPERO, number CRD42018111981.

**RESULTS:**

Among 5,244 studies, we identified 47 eligible articles that provided 228 survival estimates. Only five studies were entirely or partially conducted in low-income or middle-income countries. Five-year survival from embryonal tumors increased from 37% in 1980 to approximately 60% in 2009. Although survival for medulloblastoma improved substantially (from 29% to 73% during 1959-2009), survival for primitive neuroectodermal tumors wavered over time (1973-2009) and between countries. Five-year survival from astrocytoma changed very little over the 27 years between 1982 and 2009 (from 78% to 89%). Interpretation of the literature was made difficult by the heterogeneity of study designs.

**CONCLUSION:**

Survival has improved for embryonal tumors, but little change has been observed for astrocytic tumors. We found a striking gap in knowledge about survival from childhood brain tumor subtypes in middle-income and low-income countries, where half of these tumors are diagnosed. Larger studies are needed, including in under-represented countries and based on standardized data collection, to provide up-to-date survival estimates.

## INTRODUCTION

Primary tumors of the CNS in children are rare. The estimated world-standardized incidence rate in 2018 was 12 cases per million, ranging from 1.8 in Melanesia to 36.0 in North America.^1^ Despite their rarity, primary CNS tumors were estimated to be the second most important cause of childhood cancer–related deaths after leukemia. The estimated world-standardized mortality rate in 2018 was 0.7 deaths per million, varying between 0.04 in Tanzania and 2.4 in Honduras.^1^

Incidence and mortality are essential indicators of the cancer burden in a given population, but the duration of survival also accounts for the dynamic nature of the process between diagnosis and death. Therefore, population-based survival is the most appropriate measure to assess the overall effectiveness of a health care system in managing a given cancer.^2,3^ The third cycle of the CONCORD program (CONCORD-3) found wide disparities in survival among more than 700,000 patients who were diagnosed with a primary brain tumor in 58 countries worldwide during the 15-year period of 2000-2014. Five-year net survival for all childhood brain tumors combined ranged from 29% in Brazil to approximately 80% in several European countries.^4^ International disparities in survival may result from obstacles in access to surgery, radiotherapy, and chemotherapy.^5-8^ Such inequalities will inevitably result in failure to diagnose and treat brain tumors adequately, ultimately leading to premature deaths.^9^

CNS tumors comprise tumors of the brain, the spinal cord, and the meninges, but brain tumors are by far the largest group. Brain tumors vary widely in terms of histology and clinical behavior. Histology plays a pivotal role in treatment planning, and treatment needs are specific to each tumor subtype. Therefore, a breakdown of the observed disparities in survival by histology is warranted to help shape cancer control plans. In the fifth cycle of the EUROpean CAncer REgistry based study on survival and care of cancer patients (EUROCARE-5) study, which involved children diagnosed during 2000-2007 in 27 European countries, the average 5-year observed survival was 95% for children diagnosed with pilocytic astrocytoma and 65% for those affected by medulloblastoma. This study showed very wide international disparities. For instance, among children diagnosed with a brain tumor defined as WHO grade III or IV, 5-year survival ranged from 36% in Bulgaria to 66% in Finland.^10^

**CONTEXT**

**Key Objective**
To explore what is known about time trends and global variation in population-based survival from common childhood brain tumors.
**What Is Known**
Five-year survival from medulloblastoma increased from 23% to 73% during 1960-2010, while survival from astrocytoma (nonmalignant and malignant combined) persisted in the range of 80%-90% (1970-2010). Scarce data were available from low-income and middle-income countries, where most childhood brain tumors are currently diagnosed.
**Relevance**
Our systematic review of real-world, population-based survival estimates may inform clinicians about expected outcomes in unselected populations of children with brain tumor. The available estimates, however, do not cover countries with limited resources, where obstacles in access to care may result in suboptimal treatment. Global initiatives aiming to improve survival of children with brain tumor are underway, and they require a more recent, wide-ranging survival benchmark, which can be obtained only through larger studies using the same protocol for data collection, centralized data quality checks, and the same statistical methodology.

To our knowledge, no summary of the scientific evidence on population-based survival for the main subtypes of brain tumor in children is available. We aimed to fill this gap in knowledge by conducting the first systematic review on time trends and geographic variation in survival from brain tumors.

## PATIENTS AND METHODS

We considered longitudinal, observational studies that provided estimates of population-based survival, by histology, for children (mainly those age 0-14 years) diagnosed with a primary brain tumor, either malignant or nonmalignant. We excluded studies that only included patients with a CNS tumor in anatomic sites other than the brain because of their rarity and the paucity of data. We also excluded studies that only presented survival estimates for all histologies combined. Studies had to be based on primary data drawn from population-based cancer registries. To maximize geographic coverage, we did not discard studies presenting hospital-based estimates if those estimates were likely to be representative of a given country or territory (eg, a single referral center or a comprehensive network of referral centers) and if no population-based estimate was available. We also excluded clinical trials or clinical series, because these study designs only include selected patients. Studies were eligible if they included estimates of the survival probability from a time-to-event analysis. To improve comparability between studies, only those presenting survival estimates at 5 years after the diagnosis were included.

We searched six databases (Dissertation and Theses Global, Embase, Medline, Open Grey, Scopus, and Web of Science) from database inception to September 30, 2018, using predefined search strategies that included terms related to the disease under study, the statistical method, and the study design. A professional librarian at the London School of Hygiene and Tropical Medicine reviewed the search strategies (Appendix Table A1).

There were no restrictions relating to language or publication status. However, we excluded studies published before 1995, because the versions of the reference classifications were too early to allow comparability with subsequent editions.

**FIG 1:**
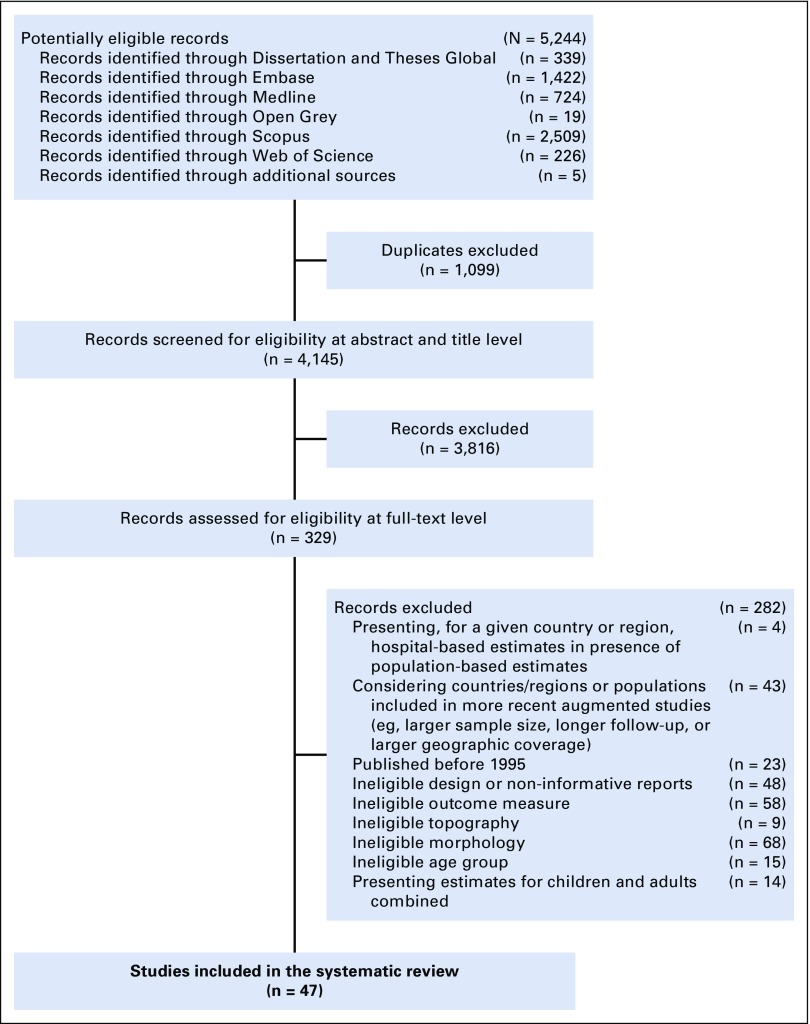
Preferred Reporting Items for Systematic Reviews and Meta-Analyses (PRISMA) flowchart.

According to the Preferred Reporting Items for Systematic Reviews and Meta-Analyses (ie, PRISMA) approach (Fig 1),^11^ potentially eligible studies were evaluated at three progressive levels: title, abstract, and full text. When eligibility was unclear, we reached an agreement through discussion.

For each eligible study, we extracted data on the tumor subtypes included and the reference classification used for tumor definitions (eg, International Classification of Diseases for Oncology, third edition [ICD-O-3]).^12^ We collected, when available, specifications of data quality indicators: the proportions of microscopically verified tumors, poorly specified/unspecified morphologies, patients lost to follow-up, and whether diagnoses based on death certificate only or autopsy were excluded. We recorded the 5-year survival probabilities for each eligible subtype and, when available, the corresponding survival estimates for each calendar period. Last, for each cancer registry, we sought information on the proportion of the population covered and on the completeness of ascertainment.

For studies considering several calendar periods, we abstracted each survival estimate separately. The calendar periods examined varied widely between studies, so we described trends by using the middle year of the corresponding time interval. Given the sparseness of data for some very rare subtypes, we focused on the most frequent morphologies, namely astrocytic and embryonal tumors. Morphologic groupings and definitions also differed between studies. We combined different definitions for the same subtype under a common descriptor (Appendix Table A2).

## RESULTS

We assessed 5,244 records for eligibility. Forty-seven studies were included in the systematic review. For each study, we detailed the following: location, completeness of ascertainment, population covered, calendar period for incident cases, age range, quality indicators available, reference classification, and outcome measure (Table 1).

**TABLE 1:**
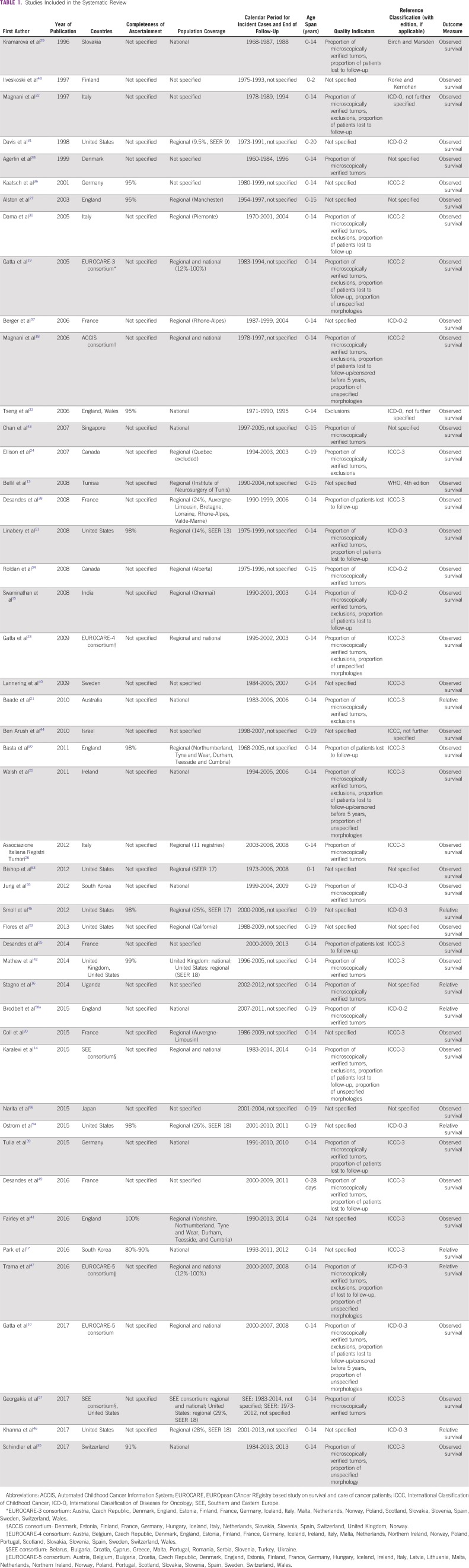
Studies Included in the Systematic Review

In thirty studies (64%), patients were age 0-14 years; they were 0-15 years in four studies and were 2 years or younger in three studies. Nine studies (19%) included individuals age 20 years or younger, and the upper age limit in one study was 24 years. Studies using nonstandard age definitions were included here, because the study populations comprised mainly children.

Nineteen studies (40%) had regional population coverage, 10 (21%) were based on nationwide registries, eight (17%) were international studies based on both regional and national registries, and the information was not available in 10 studies. Only five studies were entirely or partially conducted in low-income or middle-income countries.^13-16^ The calendar period for incident cases ranged from 1954 to 2014 (Table 1). The eligible studies collectively provided 228 survival estimates.

**FIG 2:**
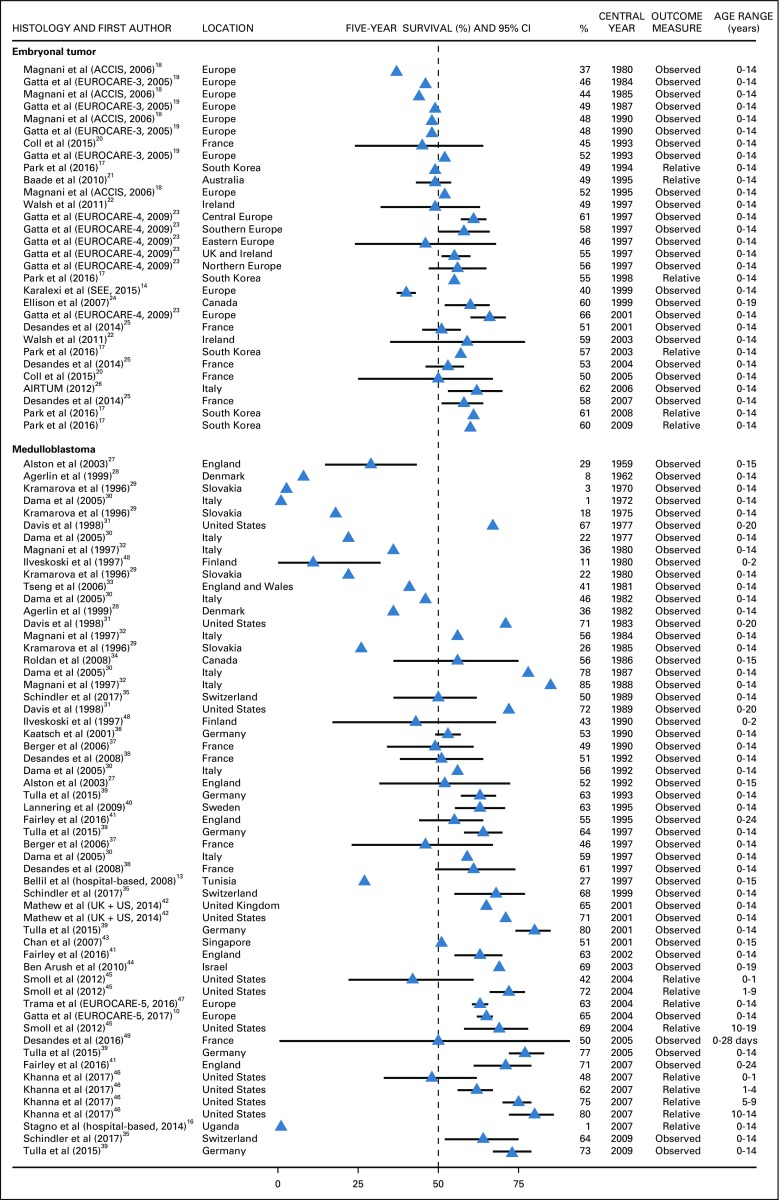
Five-year survival (%) from embryonal tumors and medulloblastoma. Automated Childhood Cancer Information System (ACCIS) consortium: Denmark, Estonia, Finland, France, Germany, Hungary, Iceland, Italy, Netherlands, Slovakia, Slovenia, Spain, Switzerland, United Kingdom (UK), Norway. EUROpean CAncer REgistry based study on survival and care of cancer patients (EUROCARE)-3 consortium: Austria, Czech Republic, Denmark, England, Estonia, Finland, France, Germany, Iceland, Italy, Malta, Netherlands, Norway, Poland, Scotland, Slovakia, Slovenia, Spain, Sweden, Switzerland, Wales. EUROCARE-4 consortium: Austria, Belgium, Czech Republic, Denmark, England, Estonia, Finland, France, Germany, Iceland, Ireland, Italy, Malta, Netherlands, Northern Ireland, Norway, Poland, Portugal, Scotland, Slovakia, Slovenia, Spain, Sweden, Switzerland, Wales. Southern and Eastern Europe (SEE) consortium: Belarus, Bulgaria, Croatia, Cyprus, Greece, Malta, Portugal, Romania, Serbia, Slovenia, Turkey, Ukraine. AIRTUM (Associazione Italiana Registri Tumori). EUROCARE-5 consortium: Austria, Belgium, Bulgaria, Croatia, Czech Republic, Denmark, England, Estonia, Finland, France, Germany, Hungary, Iceland, Ireland, Italy, Latvia, Lithuania, Malta, Netherlands, Northern Ireland, Norway, Poland, Portugal, Scotland, Slovakia, Slovenia, Spain, Sweden, Switzerland, Wales. The CI is not displayed when the study did not provide it.

For patients diagnosed with embryonal tumors as a broad histology group, 5-year survival increased substantially during the 30 years between 1980 and 2009, from 37% in 1980 to approximately 60% in 2009.^14,17-26^ In most countries, the survival probability was 50% or lower until 1997.^17-26^ Despite this positive trend, there were remarkable geographic disparities. Around 2000, there was a 26% gap in 5-year survival between the Southern and Eastern Europe (SEE) consortium, including middle-income countries such as Belarus, Bulgaria, and Ukraine (40%),^14^ and the EUROCARE-5 consortium, which includes all of the most affluent European countries (66%; Fig 2).^23^

Five-year survival from medulloblastoma increased from 29% to 73% during the 50 years between 1959 and 2009.^27-46^ In Denmark, Italy, and Slovakia, survival was 10% or less until 1972. In Denmark and Italy, survival increased sharply during the following decade (approximately 40% in 1982), while survival in Slovakia was still 26% in 1985.^28-30^ In most of the European countries, the survival probability was 60% or more after 1992^10,27,35,38-42,47^; in the United States, similar or higher values were observed in 1977.^31^ Five-year survival from medulloblastoma in Tunisia was less than 27% in 1997, and it was zero in Uganda in 2007 (n = 14 patients).^13,16^ Survival in children younger than age 2 years was 50% or lower and did not change over time (Fig 2).^45,46,48,49^

**FIG 3:**
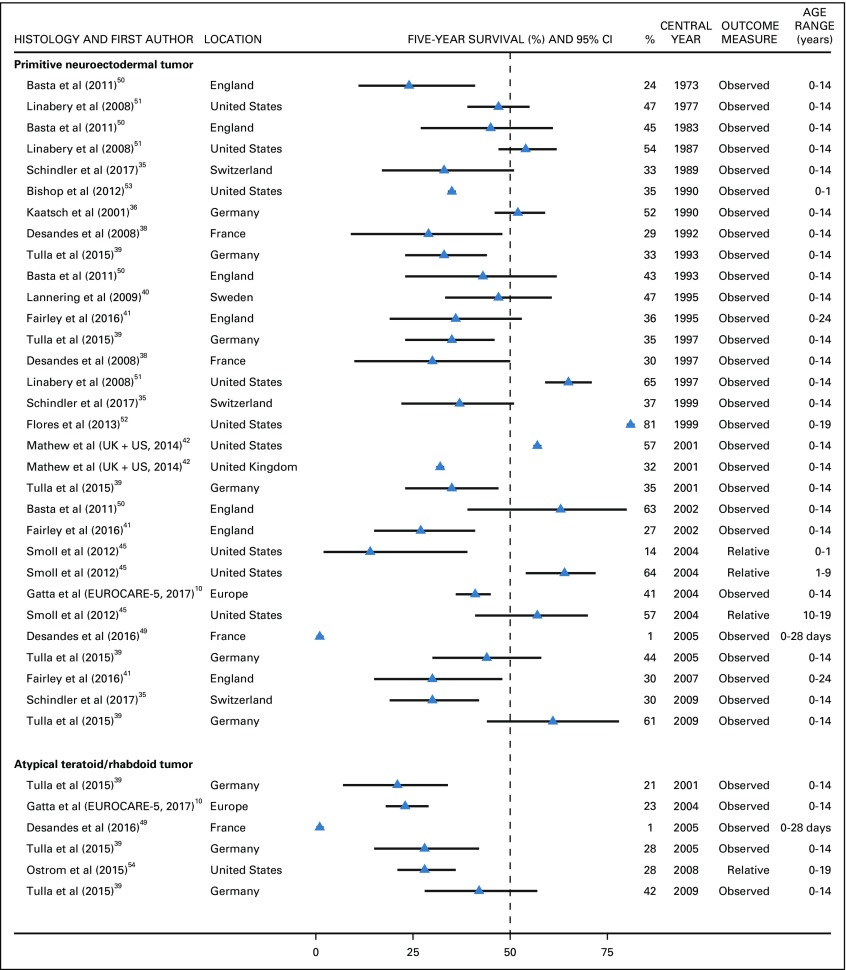
The 5-year survival (%) from primitive neuroectodermal tumor and atypical teratoid/rhabdoid tumor. EUROCARE-5 consortium: Austria, Belgium, Bulgaria, Croatia, Czech Republic, Denmark, England, Estonia, Finland, France, Germany, Hungary, Iceland, Ireland, Italy, Latvia, Lithuania, Malta, Netherlands, Northern Ireland, Norway, Poland, Portugal, Scotland, Slovakia, Slovenia, Spain, Sweden, Switzerland, Wales. The CI is not displayed when the study did not provide it. UK, United Kingdom.

Five-year survival from primitive neuroectodermal tumors (PNETs) fluctuated in the range of 27%-52% in most European countries (1973-2009) without a monotonic trend.^10,35,36,38-42,45,50-52^ In two studies conducted in England and France, survival values were not in line with those observed in other European countries, but CIs were wide.^39,50^ In the United States, 5-year survival ranged between 47% and 81% during 1977-2009.^42,45,51,52^ These values were higher than those observed in Europe during the four decades between 1973 and 2009 (24%-47%). Five-year survival from PNET in infants (age 1 year or younger) varied between zero and 33% (1990-2004), but data were scant and inconsistent (Fig 3).^45,49,53^

For children diagnosed with atypical teratoid/rhabdoid tumor, a rare subtype of embryonal tumor, 5-year survival in Germany increased from 21% to 42% during 2001-2009, but CIs overlapped.^39^ In the United States^54^ and the EUROCARE-5 consortium,^10^ the survival probability during 2004-2008 was 30% or less (Fig 3).

**FIG 4:**
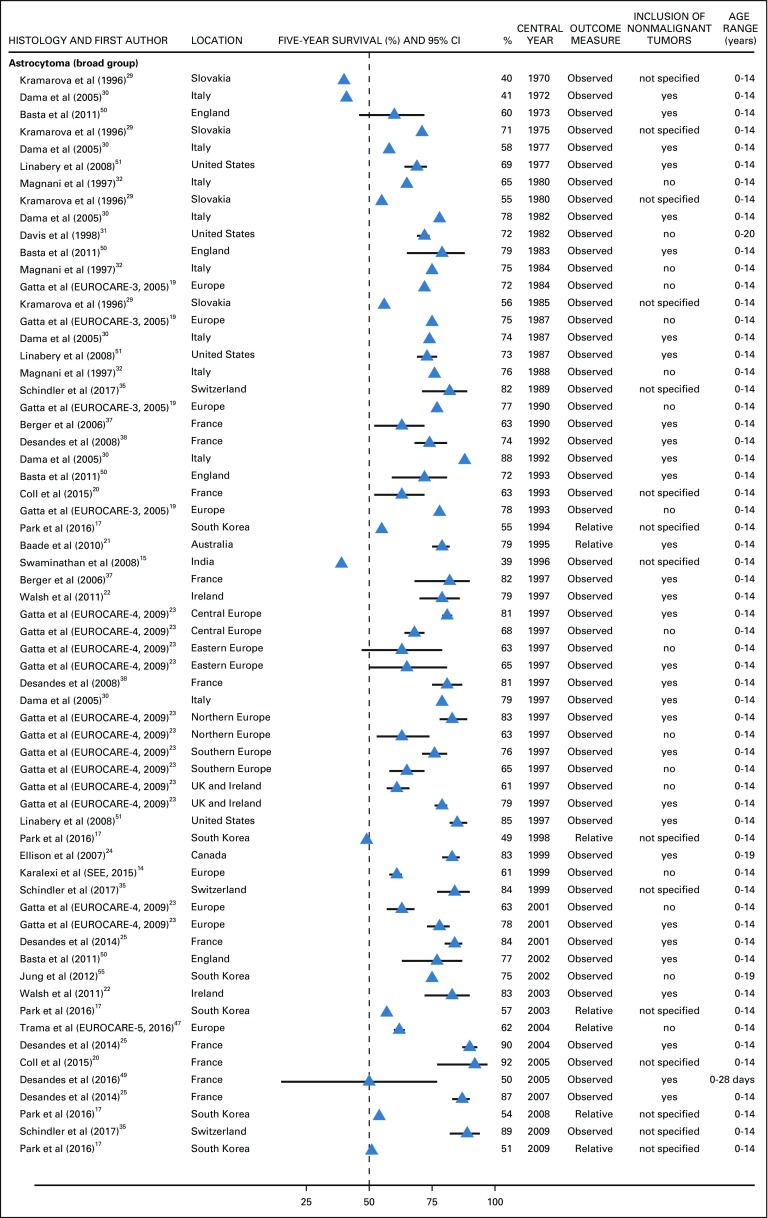
Five-year survival (%) from astrocytoma (broad group). EUROpean CAncer REgistry based study on survival and care of cancer patients (EUROCARE-3) consortium: Austria, Czech Republic, Denmark, England, Estonia, Finland, France, Germany, Iceland, Italy, Malta, Netherlands, Norway, Poland, Scotland, Slovakia, Slovenia, Spain, Sweden, Switzerland, Wales. EUROCARE-4 consortium: Austria, Belgium, Czech Republic, Denmark, England, Estonia, Finland, France, Germany, Iceland, Ireland, Italy, Malta, Netherlands, Northern Ireland, Norway, Poland, Portugal, Scotland, Slovakia, Slovenia, Spain, Sweden, Switzerland, Wales. Southern and Eastern Europe (SEE) consortium: Belarus, Bulgaria, Croatia, Cyprus, Greece, Malta, Portugal, Romania, Serbia, Slovenia, Turkey, Ukraine. EUROCARE-5 consortium: Austria, Belgium, Bulgaria, Croatia, Czech Republic, Denmark, England, Estonia, Finland, France, Germany, Hungary, Iceland, Ireland, Italy, Latvia, Lithuania, Malta, Netherlands, Northern Ireland, Norway, Poland, Portugal, Scotland, Slovakia, Slovenia, Spain, Sweden, Switzerland, Wales. The CI is not displayed when the study did not provide it. UK, United Kingdom.

Astrocytoma as a broad histology group was the most commonly adopted definition. Five-year survival was 71% or lower during 1970-1980,^29,30,32,50,51^ and it increased slightly over three decades, from 78% in 1982 to 89% in 2009.^14,17,19,20,22-25,29-32,35,37,47,50,51,55^ During 1982-1996, when the ICD-O-2 was in force,^56^ 5-year survival for astrocytoma ranged between 72% and 82% in most countries.^19,21,30-32,35,38,50,51^ In the EUROCARE-4 study^23^ (which used the ICD-O-3^12^), 5-year survival for astrocytoma (1995-2002) in Central Europe, Northern Europe, Southern Europe, the United Kingdom, and Ireland was also approximately 75% when all behaviors were considered, but it decreased by 10% when nonmalignant tumors were excluded. In Eastern Europe, the survival probability was approximately 65% regardless of tumor behavior.^23^ Similarly, in the EUROCARE-5 study, 5-year survival for malignant astrocytoma was in the range of 60%-65% during 2000-2007.^47^ Five-year survival from astrocytoma in India was 39% in 1996, whereas survival in the SEE consortium was similar to that of other European regions (Fig 4).^14,15^

**FIG 5:**
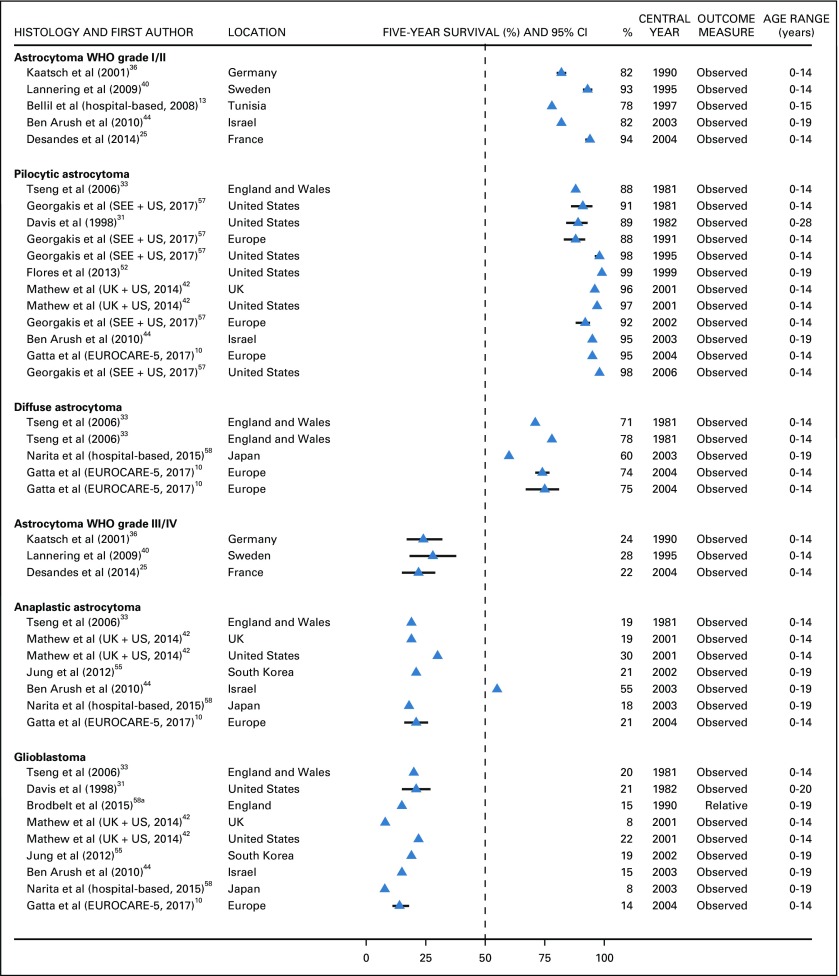
Five-year survival (%) from astrocytoma WHO grade I and II, pilocytic astrocytoma, diffuse astrocytoma, astrocytoma WHO grade III and IV, anaplastic astrocytoma, and glioblastoma. EUROpean CAncer REgistry based study on survival and care of cancer patients (EUROCARE-5) consortium: Austria, Belgium, Bulgaria, Croatia, Czech Republic, Denmark, England, Estonia, Finland, France, Germany, Hungary, Iceland, Ireland, Italy, Latvia, Lithuania, Malta, Netherlands, Northern Ireland, Norway, Poland, Portugal, Scotland, Slovakia, Slovenia, Spain, Sweden, Switzerland, Wales. The CI is not displayed when the study did not provide it. SEE, Southern and Eastern Europe; UK, United Kingdom.

Five-year survival for low-grade astrocytoma (WHO grades I and II combined) was 80% or more in Europe, the United States, and Israel,^25,36,40,44^ but it was slightly less than 80% in Tunisia.^13^ For patients diagnosed with pilocytic astrocytoma during 1981-1991, 5-year survival was in the range of 88%-91% in England, Wales, the United States, and Southeastern Europe. During 1995-2004, 5-year survival from pilocytic astrocytoma increased to 95% or more in the United States, Israel, and the EUROCARE-5 consortium,^10,42,44,52,57^ but it remained unchanged in Southeastern Europe.^57^ Five-year survival for diffuse astrocytoma was in the range of 60%-78% in Europe and Japan during 1981-2004 (Fig 5).^10,33,58^

Five-year survival for high-grade astrocytoma (WHO grades III and IV combined) was 20%-30% in France, Germany, and Sweden (1990-2004).^25,36,40^ Five-year survival probability for anaplastic astrocytoma was 30% or lower in Europe,^10,33,42^ Japan,^58^ South Korea, and the United States,^42,55^ but it was 55% in Israel.^44^ Five-year survival for glioblastoma was in the range of 8%-20% in Europe,^10,33^ Israel,^44^ Japan,^58^ South Korea, and the United States^31,42,55^ (Fig 5). For both low-grade and high-grade astrocytomas, there was no improvement in the observed outcomes during the 25 years between 1981 and 2004.

Among 47 studies, only 11 (23%) specified the completeness of case ascertainment. One third (36%) did not provide details on data quality. Twenty-six studies (55%) specified at least the proportion of microscopically verified tumors, and seven of them only included patients with microscopically verified tumors (Appendix Table A3). Four of the eight international studies specified the proportion of histologically confirmed brain tumors,^10,14,42,57^ whereas the others reported a proportion for all childhood tumors combined (Appendix Table A3).^18,19,23,47^ Seven studies (15%) did not specify the reference classification, and two did not clarify the version of the ICD-O or the International Classification of Childhood Cancer (ICCC) that was used (Table 1). Five of the 22 studies using the definition “astrocytoma” (broad histology group) did not elucidate whether they included only malignant tumors or both malignant and nonmalignant tumors (Appendix Table A3).^15,17,20,29,35^ Thirty-nine studies (83%) provided estimates of all-cause survival (ie, observed). Only eight provided relative survival estimates, adjusted for background mortality (Table 1).

## DISCUSSION

To our knowledge, this is the first systematic review synthesizing trends and geographic variation in survival for the most common morphologic subtypes of brain tumor in children. Five-year survival for embryonal tumors increased remarkably during the 1980s and the 2000s, and the change was driven mostly by an improvement in the outcome of patients diagnosed with medulloblastoma. Survival from astrocytic tumors changed very little, regardless of WHO grade.

Only five studies included patients diagnosed in low-income or middle-income countries (Belarus, Bulgaria, India, Montenegro, Romania, Serbia, Tunisia, Turkey, Uganda, Ukraine).^13-16^ In this setting, the magnitude of the survival gap depended on country and histology, albeit the largest deficit was seen for embryonal tumors. In high-income countries, where nearly all of the studies were conducted, outcomes were similar. However, in the United States, survival from the most common embryonal tumors improved earlier than elsewhere.

Low-grade gliomas represent approximately one third of all CNS tumors in children. They are biologically distinct from low-grade gliomas seen in adults, and progression to higher-grade lesions rarely occurs.^59^ Pilocytic astrocytoma is the most common glioma subtype in children.^60^ We adopted different levels of granularity in the histology definitions, but data for specified astrocytic tumors were sparse. Most studies presented survival estimates on the basis of the second tier of the ICCC, group IIIb (ie, astrocytoma).^61^ For tumors defined as astrocytoma (broad group), corresponding to the ICCC group IIIb, 5-year survival was approximately 90% during 2004-2009. During a comparable period, survival from pilocytic astrocytoma (WHO grade I) was nearly 100%. Pilocytic astrocytoma therefore is probably responsible for the favorable outcome observed in the broader group, because diffuse astrocytoma, anaplastic astrocytoma, and glioblastoma combined only constituted approximately 30% of astrocytic tumors in children.^10^

The current recommendation is to present survival in children separately for each ICCC group. Even though pilocytic astrocytoma is predominant in children, we believe that the adoption of a broad category, such as astrocytoma (ICCC-3 group IIIb), does not fully account for international variations in survival, and it may actually attenuate the observed trends and differences. We chose to report survival at 5 years, because that is the most commonly reported time landmark, and to facilitate comparisons between studies. Low-grade gliomas are often indolent tumors that progress slowly, even after partial resection or biopsy.^62^ In a large US study that included nearly 3,500 children (age 0-20 years) diagnosed with low-grade gliomas during 1973-2005, the survival probability at 10 years for WHO grade I and grade II tumors was approximately 90% and 80%, respectively.^63^ Therefore, outcomes for this cancer subtype may be better described with longer-term survival estimates.

A remarkable proportion of studies adopting the definition of astrocytoma (as a broad histology group) did not clarify the tumor behavior. This information is necessary to interpret trends correctly. In the second edition of the ICD-O (in force from 1990),^56^ pilocytic astrocytoma was coded as malignant (behavior code 3), but, in the third edition (in force from 2000), it was attributed a borderline behavior (code 1).^12^ In studies considering patients diagnosed during 1982-1996, which used ICD-O-2, survival from astrocytoma was likely to be high as a result of the inclusion of pilocytic astrocytoma, which was defined at that time as a malignant tumor. In brain tumors, location is more important than it is for tumors at other anatomic sites, because location affects clinical presentation, diagnosis, treatment, and morbidity. Therefore, though pilocytic astrocytoma was reclassified as a nonmalignant tumor in ICD-O-3, most studies published after 2000, when ICD-O-3 was adopted, included all brain tumors, regardless of behavior. As a result, survival estimates from these studies were in fact comparable to those in earlier reports that were based on ICD-O-2. In EUROCARE-5, however, survival from astrocytoma in Eastern Europe was similar, regardless of whether tumors with borderline behavior were included or not.^23^ This finding suggests under-registration of nonmalignant brain tumors in Eastern Europe.

Medulloblastoma is the most common embryonal tumor, with a peak incidence at approximately 7 years of age. Treatment includes a combination of surgery, craniospinal irradiation, and chemotherapy. In this review, the steepest gain in survival from medulloblastoma occurred before 1992, possibly reflecting improvement in radiotherapy techniques.^64^ The effect of adding chemotherapy with lomustine, cisplatin, and vincristine after radiotherapy was first assessed in a phase II trial in the 1990s.^65^ In light of the observed benefit, the use of chemotherapy became standard. In the 1990s and 2000s, 5-year survival increased from approximately 60% to 70%. This finding may be the joint result of improved surgical management and incorporation of chemotherapy into routine clinical practice.^66^ Survival from medulloblastoma was much lower in low-income and middle-income countries than in high-income countries. This disparity may reflect the lack of access to optimal multimodality treatment.^5-8^

In three studies, medulloblastoma was grouped with PNETs, even though ICD-O-3 was given as the reference classification.^50-52^ As a result, survival estimates were higher than those for PNET only.^35,38,41,42^ Infratentorial medulloblastoma and supratentorial PNET are distinct entities, described as separate morphologies in the second edition of the WHO classification of CNS tumors (2002).^67^ Because medulloblastoma has a more favorable outcome than PNET, its inclusion in a wider group mislabeled as PNET will bias the survival estimates upward.

Two studies defined astrocytoma, not otherwise specified (NOS), as a separate morphologic entity, perhaps to allow for a generic diagnosis of unspecified astrocytic tumor.^10,33^ In the United States, the proportion of astrocytic tumors registered as astrocytoma NOS decreased from 47% to 13% during 1973-2005.^63^ The WHO classification does not recognize astrocytoma NOS as a distinct definition. Diffuse astrocytoma and astrocytoma NOS share the same ICD-O-3 code, but the WHO classification retains only the first of the two descriptors.^12,60^ Therefore, we grouped together the survival estimates, which proved comparable (70%-80%).^10,33^

In most of the studies reviewed here, indicators of data quality were often missing or incomplete. The proportion of tumors that had been microscopically verified was the most widely available parameter. Few studies reported any additional descriptors, such as the proportion of patients who were lost to follow-up before the end of the study. The proportion of microscopically verified tumors pertains not only to disease management, namely whether surgery or biopsy was performed, but also to whether the cancer registry had access to pathology reports.^68^ The proportions of microscopically verified brain tumors were in the range of 73%-93% in the SEE consortium (1983-2014) and 71%-100% in the EUROCARE-5 study (2000-2007).^10,14^

The proportion of brain tumors that are microscopically verified is typically lower than for other types of cancer, because brain tumors are more lethal and patients are often too unwell to undergo an invasive diagnostic procedure; advanced surgical expertise also is required. If the proportion of tumors that are histologically unclassified is high, survival estimates for specific morphologies may be biased, because patients with histologically confirmed disease are likely to have higher survival than those whose tumors could not be pathologically confirmed.

Similarly, information on the completeness of ascertainment of brain tumors was very often missing. In most of the studies for which this information was available, it was usually reported as nearly complete (95% or more). This parameter is important to assess whether the patients included in the study are representative of all patients with brain tumors in the population of a given region or country.^69^

In most of the studies (83%), survival was reported only as observed survival, without taking into account death as a result of causes other than the brain tumor (background mortality). If competing risks of death are not properly accounted for, survival estimates will be biased downward. Background mortality also varies widely between countries and over time, so valid international comparisons require that background mortality is incorporated in the survival estimates. However, nearly all of the studies were conducted in affluent countries, where background mortality in children has generally been very low for several decades. The distortion in international comparisons of brain tumor survival in children is thus likely to be small.

This systematic review was affected by several limitations. First, we aimed to give a comprehensive account of variations in brain tumor survival by including all of the relevant histology categories. However, very few studies were available for some categories, precluding robust conclusions on time trends and geographic variations in survival. Second, almost all the studies were based on regional rather than national data. Assuming that regional survival estimates are applicable to the whole country may not be wise in the presence of regional disparities in access to or provision of treatment within a given country. However, data from most of these regions were later included in wider studies with national or international coverage. Survival estimates from those studies were in line with those previously reported at regional level, suggesting that findings from the earlier, smaller studies were indeed generalizable to the country. Finally, the dates and the length of calendar periods in which the patients had been diagnosed also varied widely between studies. To allow an orderly presentation of time trends, we referred to the central year for any given time interval, but we were not able to compare the average annual increment or decrement in survival between calendar periods of different, and often overlapping, lengths. Improvements in survival were nevertheless limited mainly to embryonal tumors, and they occurred over an extended period, so the international comparisons may be considered reasonably informative.

In conclusion, there is a staggering gap in evidence about survival from the most common types of childhood brain tumor in low-income and middle-income countries. Interpretation of the literature is hampered by the very wide heterogeneity between the designs of the various studies and by the quality of the available data.

We highlight the fact that the ICCC does not allow accurate description of variation in survival from astrocytic tumors, because it does not encompass stratification by grade. The goal of the WHO Global Initiative for Childhood Cancer is to improve survival worldwide for six cancer subtypes, including low-grade gliomas.^70^ In the context of brain tumors, future assessment of the progress of this global effort will require that an informative, up-to-date survival benchmark for low-grade gliomas is set. Ultimately, the ICCC should be revised.

The 2016 WHO classification of Tumors of the Central Nervous System has redefined or replaced several diagnostic entities or subgroups by incorporating molecular classifiers.^71^ For instance, PNET is no longer included in the diagnostic dictionary, and medulloblastoma is now genetically defined. Future comparisons of survival will have to account for these changes, but capacity-building and resources are needed to extend the use of this classification, both in clinical practice and in cancer registries, especially in low-income and middle-income countries.

Larger international studies that include currently under-represented countries are warranted, and robust survival estimates are only possible through use of the same protocol for data collection, centralized and stringent data quality checks, and application of the same statistical methodology—including appropriate life tables to correct for the risk of death as a result of causes other than cancer.

## Figures and Tables

**TABLE A1:**
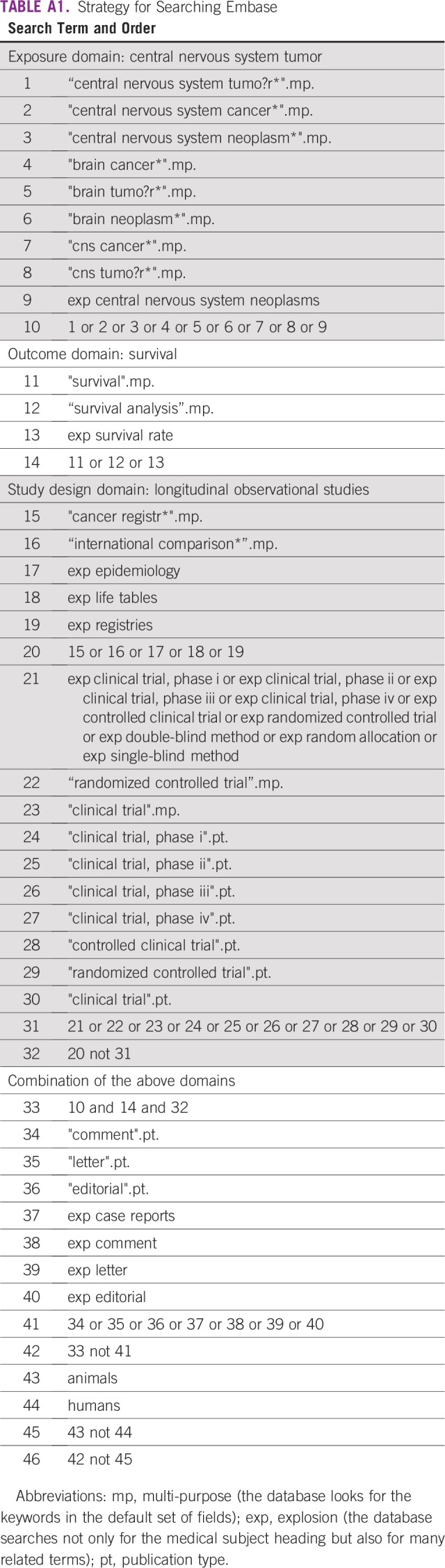
Strategy for Searching Embase

**TABLE A2:**
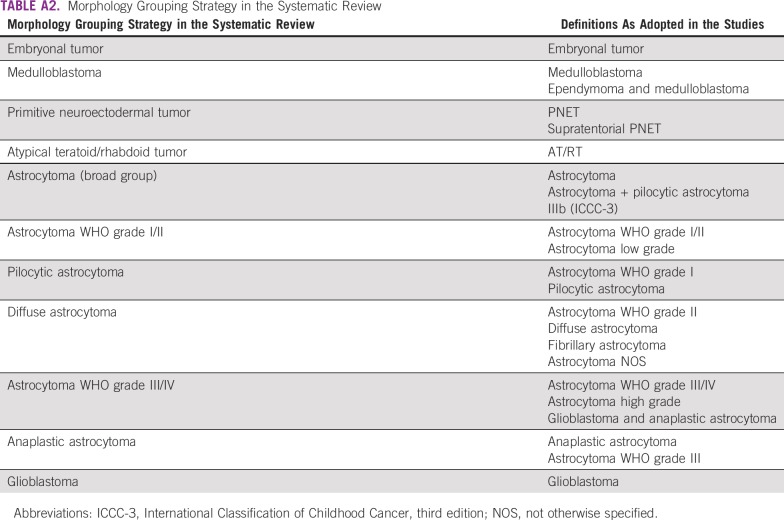
Morphology Grouping Strategy in the Systematic Review

**TABLE A3:**
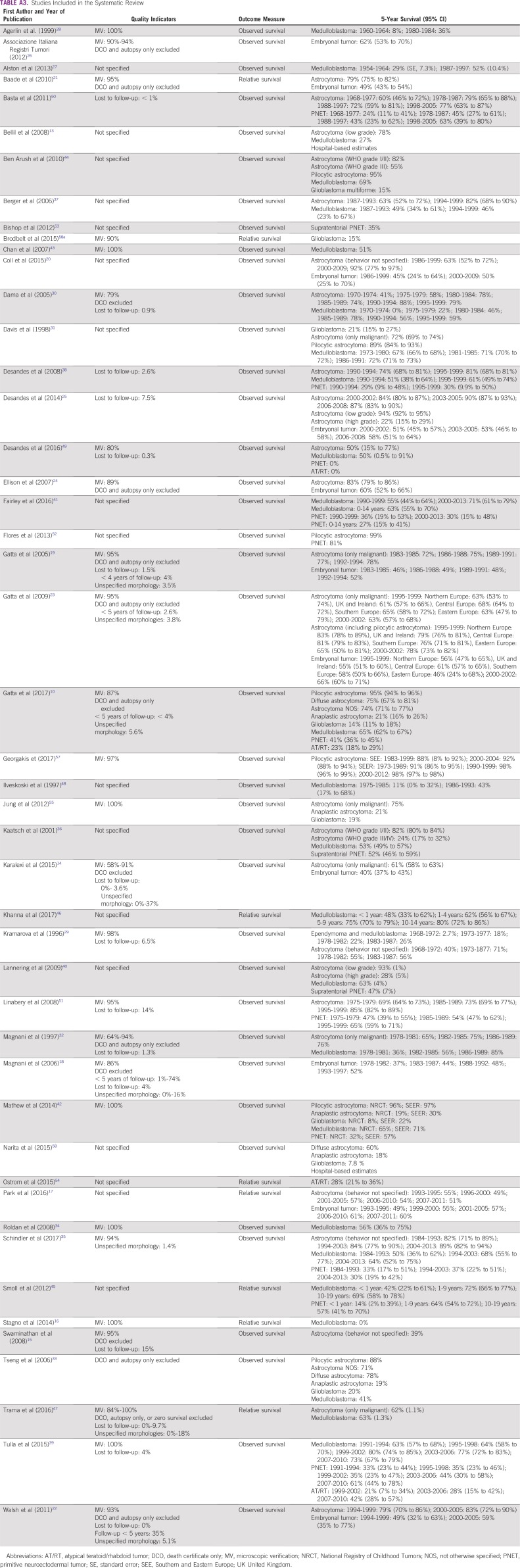
Studies Included in the Systematic Review
